# Overweight or obesity and their association with cardiometabolic risk factors among oilfield workers in Chinese population: a cross-sectional study

**DOI:** 10.3389/fpubh.2026.1658235

**Published:** 2026-02-16

**Authors:** Xuefeng Yu, Sicheng Zhang, Haobiao Liu, Qingsong Li, Yujie He, Lianxu Jia, Zhiyong Du, Jing Han

**Affiliations:** 1 Department of Occupational and Environmental Health, School of Public Health, Health Science Center, Xi'an Jiaotong University, Xi'an, China; 2 The Second Clinical Medical College of Xi'an Jiaotong University, Xi'an, China; 3 Department of Health Management, Xi'an Gem Flower Changqing Hospital, Xi'an, China

**Keywords:** body mass index, cardiometabolic risk factors, obesity, occupational health, oilfield workers

## Abstract

**Objectives:**

Overweight and obesity are major factors associated with cardiometabolic disorders, yet evidence from occupational populations remains limited. This study aimed to assess the association between body mass index (BMI) and six cardiometabolic risk factors (CRFs)—hypertension, diabetes, high total cholesterol (TC), high triglycerides (TG), high low-density lipoprotein cholesterol (LDL-C), and low high-density lipoprotein cholesterol (HDL-C)—among Chinese oilfield workers.

**Methods:**

A cross-sectional analysis was conducted among 3,048 participants undergoing routine health examinations in 2022. BMI was categorized using Chinese-specific criteria. Logistic regression models and restricted cubic spline (RCS) analyses were used to evaluate associations between BMI and six CRFs. Subgroup analyses were conducted for each of the six CRFs across pre-specified effect modifiers (age, sex, chemical exposure, night-shift work, and smoking status), and sensitivity analyses were also performed.

**Results:**

Overweight and obesity were associated with higher odds of all CRFs, with obesity showing the strongest associations. In fully adjusted models, obesity was significantly associated with higher odds of hypertension (OR = 7.08), diabetes (OR = 3.25), high TC (OR = 1.81), high TG (OR = 4.80), high LDL-C (OR = 3.07), and low HDL-C (OR = 3.36) compared to normal participants. RCS analyses revealed dose–response pattern, with nonlinearity for high TG and low HDL-C (both *p* < 0.001). Subgroup analyses showed generally consistent associations, with stronger associations observed for hypertension among current smokers, for diabetes in older participants, and for low HDL-C in male. Sensitivity analyses supported the robustness of these findings.

**Conclusion:**

BMI was independently associated with higher odds of hypertension, diabetes, and dyslipidemia, with obesity exhibiting the strongest associations across CRFs. These findings underscore the relevance of excess adiposity in relation to early metabolic abnormalities and may help inform occupational health monitoring strategies.

## 1 Introduction

Overweight and obesity, defined as abnormal or excessive fat accumulation, have become increasingly prevalent across the globe (1). They have emerged as critical public health challengesand are strongly associated with the global burden of non-communicable diseases and premature mortality (2, 3). In China, these conditions have surged in recent decades, driven by rapid urbanization, shifts toward high-energy diets, and sedentary lifestyles (4). Projections suggest that by 2030, approximately 70.5% of Chinese adults will be classified as overweight or obesity (5). This rising prevalence has serious implications for public health, particularly due to its strong association with cardiovascular and metabolic disorders (6, 7). According to the 2023 Report on Cardiovascular Health and Diseases in China, cardiovascular diseases (CVDs)—often linked to obesity and its metabolic complications—accounted for 47.35% of all urban and 48.98% of all rural deaths in 2021 (4). These trends underscore the urgency of understanding obesity’s association with the presence of cardiometabolic disorders. Given their unique occupational exposures and lifestyle patterns, oilfield employees represent a key but understudied population for investigating the early manifestations of obesity-related metabolic risk.

Cardiometabolic risk factors (CRFs) refer to a constellation of measurable physiological indicators—including elevated blood pressure, impaired fasting glucose, high triglycerides, high low-density lipoprotein cholesterol (LDL-C), and low high-density lipoprotein cholesterol (HDL-C)—that individually or collectively increase the odds of CVDs (8). These factors are strongly associated with indicators of atherosclerotic progression, endothelial dysfunction, and systemic inflammation (9). Increasing epidemiological evidence highlights that metabolic abnormalities have been associated with early emergence and co-occurrence of cardiometabolic risk factors—often in individuals without diagnosed disease—posing a significant health burden, especially in working populations undergoing routine health surveillance (10–13). Moreover, in the Chinese population, growing evidence suggests that cardiometabolic risk has been observed comparatively lower body mass index (BMI) thresholds, due to increased visceral adiposity and unique fat distribution patterns (14). This ethnic susceptibility highlights the need for population-specific research on the interplay between excess adiposity and CRFs, particularly in non-clinical and working populations.

Despite the well-established relationship between obesity and cardiometabolic disorders, limited data exist on how excess weight, as defined by BMI, relates to CRFs in occupational populations in China. Oilfield workers, who are often exposed to physically demanding work, irregular schedules, and suboptimal dietary patterns, may show different patterns of metabolic abnormalities. However, few studies have focused on this group or systematically evaluated how BMI categories—normal weight, overweight, and obesity—correspond to CRFs in this setting.

To address this gap, the present study aims to evaluate the associations between BMI and multiple CRFs among oilfield employees in China. By positioning BMI—a widely accepted anthropometric indicator of adiposity—as the primary exposure, and CRFs as clinically relevant early metabolic abnormalities, this study provides occupation-specific evidence on how overweight and obesity are associated with the presence of metabolic risk. Focusing on a working population undergoing routine health examinations allows for tailored insights into the role of overweight and obesity in early cardiometabolic disruption, thereby informing targeted prevention strategies and workplace health management in high-risk labor groups.

## 2 Materials and methods

### 2.1 Study setting and participants

**Figure 1:**
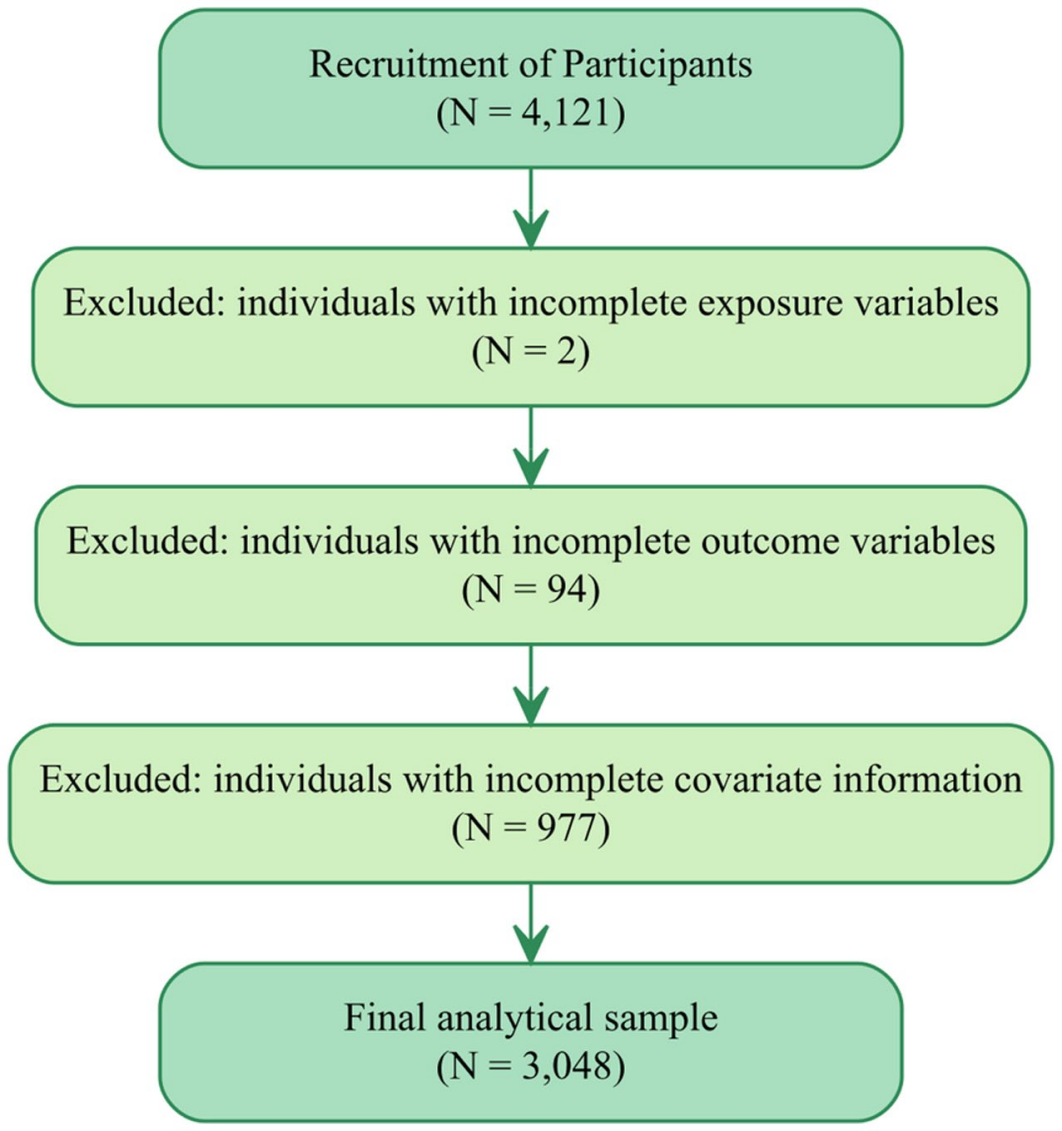
Flowchart of participant selection in the study.

A total of 4,121 individuals who underwent routine health examinations at the Xi’an Gem Flower Changqing Hospital between October 2022 to December 2022, were initially enrolled in the study. After applying predefined inclusion and exclusion criteria (Figure 1), eligible participants were invited to complete a structured questionnaire to collect additional information, including sociodemographic characteristics, lifestyle factors, occupational exposures, and self-reported history of hypertension, diabetes, and other chronic diseases. The annual health examination is a mandatory component of the occupational health management system for oilfield employees, and participation in the examination program is routinely very high (typically >95%). Accordingly, the initial sample of 4,121 workers represents nearly the entire population attending the 2022 examination cycle. The study site is located in an urban oilfield-affiliated hospital in northwest China, primarily serving employees and retirees of the petrochemical industry.

Figure 1 presents the participant selection flowchart. Eligible participants were those aged 18 years or older, not pregnant, free from severe psychiatric disorders, and who provided informed consent. Initially, 4,121 individuals were enrolled. After excluding 2 participants with missing exposure data (0.05%), 94 with incomplete outcome information (2.3%), and 977 lacking complete covariate records (23.7%), a total of 3,048 subjects met all inclusion criteria and were included in the final analysis.

### 2.2 Measurement of BMI and CRFs

Basic demographic and anthropometric data—including height, and weight—were obtained from the hospital’s health examination registry.

BMI was calculated as weight in kilograms divided by height in meters squared and categorized based on the widely used Chinese-specific standards: <24.0 kg/m^2^ as normal weight, 24.0–27.9 kg/m^2^ as overweight, and ≥28.0 kg/m^2^ as obesity. This classification reflects the specific body composition and health risk characteristics of the Chinese population and is widely used in national epidemiological studies (15), providing a more relevant assessment for our study design. Blood pressure was measured on the right arm in a seated position after at least 5 min of rest using an automated sphygmomanometer (SK-E600A, China); the mean of two readings taken one minute apart was recorded. All participants underwent venous blood sampling in the morning after an overnight fast of at least 10 h. Biochemical analyses were performed at the hospital’s physical examination department using standardized procedures. Measured indicators included fasting blood glucose (FBG), total cholesterol (TC), triglycerides (TG), LDL-C, and HDL-C.

The study considered six CRFs as outcome variables, based on widely used diagnostic thresholds in Chinese adult populations (16). Hypertension was defined as systolic blood pressure ≥140 mmHg and/or diastolic blood pressure ≥90 mmHg, or a self-reported physician diagnosis. Diabetes was defined as FBG ≥ 7.0 mmol/L or a self-reported history of diabetes. High TC was defined as TC ≥ 6.2 mmol/L, high TG as TG ≥ 2.3 mmol/L, and high LDL-C as LDL-C ≥ 4.1 mmol/L. Low HDL-C was defined as HDL-C < 1.0 mmol/L.

In this study, BMI was designated as the primary exposure variable to represent overall adiposity, given its widespread use in epidemiological research and occupational health surveillance. This anthropometric measure facilitates the assessment of overweight and obesity as a key determinant potentially related to the association with cardiometabolic factors among oilfield workers.

### 2.3 Covariates

In this study, covariates included in the adjusted models were: age, sex, education, marital status, income, ethnicity, smoking status, drinking status, exercise, night shift, chemical exposure, noise exposure and dust exposures. The categories and definitions of the covariates are shown in Supplementary Table S1.

### 2.4 Statistical analysis

Because the proportion of missing data for the primary exposure BMI and outcomes CRFs was very low (0.05 and 2.3%), complete-case analysis was chosen as the primary analytic approach. Sensitivity analyses using multiple imputation for missing covariates were performed to assess the robustness of the results.

Continuous variables are presented as mean values with standard deviation and compared among groups using analysis of variance (ANOVA). Categorical variables are reported as counts and percentages, and differences were compared using chi-square tests. We additionally performed p-for-trend analyses across BMI categories for all continuous and ordinal variables to assess potential dose–response patterns. Given the cross-sectional design of this study, logistic regression was applied to estimate odds ratios (ORs), which quantify the strength of association between BMI categories and each CRF. Because several CRFs exhibited relatively high prevalence in this population, the ORs should not be interpreted as prevalence ratios or risk ratios. In addition, due to the cross-sectional nature of the data, the temporal direction of the associations cannot be established, and reverse causation cannot be excluded. Logistic regression was selected to ensure comparability with previous occupational and epidemiologic studies evaluating BMI–CRF associations. Three logistic regression models were used to estimate ORs and 95% CIs for the associations between BMI and CRFs. Model 1 included no covariate adjustment. Model 2 was adjusted for age, sex, education, marital status, income, and ethnicity. Model 3 included additional covariates—exercise, smoking status, drinking status, night shift, chemical exposure, noise exposure, and dust exposure—that may act as confounders or contextual modifiers. These were added to evaluate the robustness of the associations under broader real-world conditions.

Restricted cubic spline (RCS) analysis was applied to evaluate potential dose–response pattern. To flexibly model the non-linear association between BMI and each CRF, RCS analysis was conducted with three knots placed at the 10th, 50th, and 90th percentiles of BMI. This configuration was chosen based on Harrell’s recommendations, balancing model flexibility and stability. Spline modeling was performed using the rms package in R (version 4.5.0), as described in Regression Modeling Strategies (17). For plotting the dose–response relationship, the BMI value corresponding to the median (50th percentile) was used as the reference, where the odds ratio was set to 1. The inflection points reported in the RCS analyses were exploratory and emerged directly from the shape of the fitted spline curves. These values were identified visually as the BMI ranges where the slope of the spline function increased or decreased more sharply, rather than through any formal statistical test or pre-specified clinical thresholds. Accordingly, these inflection points should be interpreted as descriptive features of the modeled dose–response association rather than definitive physiological or diagnostic cutoffs.

Subgroup analyses were conducted to evaluate whether the associations between BMI and CRFs differed across participant characteristics. Specifically, stratified analyses were performed by age, sex, chemical exposure, night-shift work, and smoking status, which were pre-specified *a priori* based on prior epidemiologic and occupational-health evidence suggesting their potential role as association modifiers (18–20). This pre-specification complies with reporting guidelines that distinguish planned from exploratory subgroup analyses. In these analyses, the six CRFs were examined separately, consistent with the main analyses. Fully adjusted logistic regression models were applied in each stratum, and multiplicative interaction terms were used to formally test for association modification. This approach ensured interpretability and comparability with the primary results while avoiding post-hoc exploratory analyses. Although these subgroup analyses were pre-specified, they were still considered exploratory in terms of statistical inference; therefore, no formal multiplicity correction was applied, and all subgroup findings should be interpreted as hypothesis-generating.

To assess the robustness of our findings, we performed sensitivity analyses that included multiple imputations for missing covariates using multivariate imputation by chained equations MICE with 5 imputed datasets. The results from the imputed datasets were pooled using Rubin’s rules. Additionally, we conducted a re-analysis using the alternative World Health Organization (WHO) BMI classification criteria (normal weight <25 kg/m^2^, overweight 25–29.9 kg/m^2^, and obesity ≥30 kg/m^2^). R software was used to conduct all the statistical analyses. A two-sided *p*-value < 0.05 was considered statistically significant.

## 3 Result

### 3.1 Participant characteristics

Baseline characteristics of participants by BMI category are summarized in Table 1. Among the 3,048 individuals included, 1,435 were classified as normal, 1,141 as overweight, and 472 as obesity. Significant differences across BMI groups were observed in age, sex, marital status, smoking status, drinking status, night shift and cardiometabolic conditions (all *p* < 0.01). The obesity exhibited the most pronounced disparities, with 82.42% male participants and 49.15% current smokers, compared to 79.14 and 45.14% in the overweight, and 48.57 and 28.43% in the normal, respectively. Importantly, the overweight also demonstrated significantly higher proportions than the normal (all *p* < 0.001). The prevalence of all six CRFs were significantly higher in both overweight and obesity. No significant differences were found in education, income, ethnicity, physical activity, or occupational exposures (*p* > 0.05). In Table 1, p-for-trend tests across BMI categories are presented for all continuous and ordinal characteristics. Significant increasing or decreasing trends across BMI categories were observed for sex, night shift, smoking status, drinking status, hypertension, diabetes, high TC, high TG, high LDL-C, and low HDL-C (all *p* < 0.05).

**Table 1:** Basic characteristics of the participants.

Characteristic	BMI category				*p* value	*P* for trend
	Overall (*N* = 3,048)	Normal (*N* = 1,435)	Overweight (*N* = 1,141)	Obesity (*N* = 472)		
Age, year					<0.001	0.106
20–35 years	754 (24.74%)	386 (26.90%)	237 (20.77%)	131 (27.75%)		
35–50 years	1,840 (60.37%)	870 (60.63%)	688 (60.30%)	282 (59.75%)		
≥50 years	454 (14.90%)	179 (12.47%)	216 (18.93%)	59 (12.50%)		
Sex					<0.001	<0.001
Female	1,059 (34.74%)	738 (51.43%)	238 (20.86%)	83 (17.58%)		
Male	1,989 (65.26%)	697 (48.57%)	903 (79.14%)	389 (82.42%)		
Education					0.094	0.223
High school and below	1,110 (36.42%)	495 (34.49%)	447 (39.18%)	168 (35.59%)		
College	1,069 (35.07%)	522 (36.38%)	371 (32.52%)	176 (37.29%)		
Undergraduate and above	869 (28.51%)	418 (29.13%)	323 (28.31%)	128 (27.12%)		
Marital status					<0.001	0.561
Single/divorced/widowed	515 (16.90%)	260 (18.12%)	149 (13.06%)	106 (22.46%)		
Married	2,533 (83.10%)	1,175 (81.88%)	992 (86.94%)	366 (77.54%)		
Income, thousand (CNY)					0.065	0.965
≤100	634 (20.80%)	318 (22.16%)	213 (18.67%)	103 (21.82%)		
101–150	1,952 (64.04%)	922 (64.25%)	738 (64.68%)	292 (61.86%)		
≥151	462 (15.16%)	195 (13.59%)	190 (16.65%)	77 (16.31%)		
Ethnicity					0.742	0.460
Han	2,991 (98.13%)	1,411 (98.33%)	1,118 (97.98%)	462 (97.88%)		
Other	57 (1.87%)	24 (1.67%)	23 (2.02%)	10 (2.12%)		
Exercise					0.137	0.176
Inactive	1,170 (38.39%)	558 (38.89%)	450 (39.44%)	162 (34.32%)		
Active	1,878 (61.61%)	877 (61.11%)	691 (60.56%)	310 (65.68%)		
Smoking status					<0.001	<0.001
Non-smoker	1,661 (54.49%)	952 (66.34%)	517 (45.31%)	192 (40.68%)		
Former smokers	232 (7.61%)	75 (5.23%)	109 (9.55%)	48 (10.17%)		
Current smokers	1,155 (37.89%)	408 (28.43%)	515 (45.14%)	232 (49.15%)		
Drinking status					<0.001	<0.001
Non-drinker	1,964 (64.44%)	1,052 (73.31%)	651 (57.06%)	261 (55.30%)		
Former drinkers	190 (6.23%)	61 (4.25%)	83 (7.27%)	46 (9.75%)		
Current drinkers	894 (29.33%)	322 (22.44%)	407 (35.67%)	165 (34.96%)		
Night shift					0.004	0.002
No	1,399 (45.90%)	613 (42.72%)	553 (48.47%)	233 (49.36%)		
Yes	1,649 (54.10%)	822 (57.28%)	588 (51.53%)	239 (50.64%)		
Chemical exposure					0.920	0.686
No	697(22.87%)	332(23.14%)	260(22.79%)	105(22.25%)		
Yes	2,351 (77.13%)	1,103 (76.86%)	881 (77.21%)	367 (77.75%)		
Noise exposure					0.438	0.217
No	1,146(37.60%)	552(38.47%)	428(37.51%)	166(35.17%)		
Yes	1,902 (62.40%)	883 (61.53%)	713 (62.49%)	306 (64.83%)		
Dust exposure					0.413	0.278
No	2,289(75.10%)	1,085 (75.61%)	861 (75.46%)	343 (72.67%)		
Yes	759 (24.90%)	350 (24.39%)	280 (24.54%)	129 (27.33%)		
Hypertension					<0.001	<0.001
No	2,353 (77.20%)	1,269 (88.43%)	843 (73.88%)	241 (51.06%)		
Yes	695 (22.80%)	166 (11.57%)	298 (26.12%)	231 (48.94%)		
Diabetes					<0.001	<0.001
No	2,854 (93.64%)	1,378 (96.03%)	1,068 (93.60%)	408 (86.44%)		
Yes	194 (6.36%)	57 (3.97%)	73 (6.40%)	64 (13.56%)		
High TC					<0.001	<0.001
No	2,494 (81.82%)	1,224 (85.30%)	915 (80.19%)	355 (75.21%)		
Yes	554 (18.18%)	211 (14.70%)	226 (19.81%)	117 (24.79%)		
High TG					<0.001	<0.001
No	1755 (57.58%)	1,078 (75.12%)	521 (45.66%)	156 (33.05%)		
Yes	1,293 (42.42%)	357 (24.88%)	620 (54.34%)	316 (66.95%)		
High LDL-C					<0.001	<0.001
No	2,591 (85.01%)	1,300 (90.59%)	945 (82.82%)	346 (73.31%)		
Yes	457 (14.99%)	135 (9.41%)	196 (17.18%)	126 (26.69%)		
Low HDL-C					<0.001	<0.001
No	1934 (63.45%)	1,005 (70.03%)	694 (60.82%)	235 (49.79%)		
Yes	1,114 (36.55%)	430 (29.97%)	447 (39.18%)	237 (50.21%)		

BMI, body mass index; CRFs, cardiometabolic risk factors; TC, total cholesterol; TG, triglyceride; LDL-C, low-density lipoprotein; HDL-C, high-density lipoprotein.

### 3.2 Association between BMI and cardiometabolic risk factors

As shown in Table 2, increasing BMI was positively associated with elevated odds of all CRFs, exhibiting a clear dose–response relationship across normal, overweight, and obesity categories (all *P* for trend < 0.001).

**Table 2:** Associations between body mass index and cardiometabolic risk factors.

Variable	Model 1		Model 2		Model 3	
	OR (95% CI)	*P* value	OR (95% CI)	*P* value	OR (95% CI)	*P* value
Hypertension						
Normal	1.00 (Reference)		1.00 (Reference)		1.00 (Reference)	
Overweight	2.70 (2.20, 3.34)	<0.001	2.21 (1.78, 2.76)	<0.001	2.20 (1.76, 2.75)	<0.001
Obesity	7.33 (5.76, 9.35)	<0.001	6.97 (5.39, 9.05)	<0.001	7.08 (5.46, 9.20)	<0.001
*P* for trend		<0.001		<0.001		<0.001
Diabetes						
Normal	1.00 (Reference)		1.00 (Reference)		1.00 (Reference)	
Overweight	1.65 (1.16, 2.37)	0.006	1.26 (0.87, 1.83)	0.219	1.26 (0.87, 1.84)	0.219
Obesity	3.79 (2.61, 5.52)	<0.001	3.28 (2.22, 4.88)	<0.001	3.25 (2.19, 4.84)	<0.001
*P* for trend		<0.001		<0.001		<0.001
High TC						
Normal	1.00 (Reference)		1.00 (Reference)		1.00 (Reference)	
Overweight	1.43 (1.17, 1.76)	<0.001	1.33 (1.07, 1.65)	0.010	1.32 (1.07, 1.65)	0.011
Obesity	1.91 (1.48, 2.46)	<0.001	1.80 (1.38, 2.35)	<0.001	1.81 (1.38, 2.35)	<0.001
*P* for trend		<0.001		<0.001		<0.001
High TG						
Normal	1.00 (Reference)		1.00 (Reference)		1.00 (Reference)	
Overweight	3.59 (3.04, 4.25)	<0.001	2.71 (2.28, 3.23)	<0.001	2.72 (2.28, 3.25)	<0.001
Obesity	6.12 (4.89, 7.68)	<0.001	4.70 (3.72, 5.96)	<0.001	4.80 (3.79, 6.10)	<0.001
*P* for trend		<0.001		<0.001		<0.001
High LDL-C						
Normal	1.00 (Reference)		1.00 (Reference)		1.00 (Reference)	
Overweight	2.00 (1.58, 2.53)	<0.001	1.74 (1.37, 2.23)	<0.001	1.74 (1.36, 2.22)	<0.001
Obesity	3.51 (2.68, 4.60)	<0.001	3.07 (2.32, 4.06)	<0.001	3.07 (2.32, 4.07)	<0.001
*P* for trend		<0.001		<0.001		<0.001
Low HDL-C						
Normal	1.00 (Reference)		1.00 (Reference)		1.00 (Reference)	
Overweight	1.51 (1.28, 1.77)	<0.001	2.02 (1.69, 2.42)	<0.001	2.03 (1.69, 2.43)	<0.001
Obesity	2.36 (1.91, 2.92)	<0.001	3.35 (2.66, 4.22)	<0.001	3.36 (2.67, 4.25)	<0.001
*P* for trend		<0.001		<0.001		<0.001

Model 1, no covariate was adjusted. Model 2, adjusted for age, sex, education, marital status, income, ethnicity. Model 3, adjusted for age, sex, education, marital status, income, ethnicity, exercise, smoking status, drinking status, night shift, chemical exposure, noise exposure and dust exposure. BMI, Body Mass Index; CRFs, Cardiometabolic Risk Factors; OR, odds ratio; CI, confidence interval; TC, total cholesterol; TG, triglyceride; LDL**-**C, low-density lipoprotein; HDL**-**C, high-density lipoprotein.

In unadjusted models (Model 1), BMI showed strong positive associations with all six CRFs. Obesity was associated with substantially higher odds of several CRFs, including an OR of 7.33 for hypertension, 3.79 for diabetes, and 2.36 for low HDL-C. Similarly, overweight was significantly associated with elevated odds of most CRFs, with an OR of 2.70 for hypertension.

After adjusting for demographic variables including age, sex, education, marital status, income, and ethnicity (Model 2), association estimates generally attenuated, suggesting potential confounding by population characteristics. For instance, the association between overweight and diabetes became non-significant (OR = 1.26, *p* = 0.219), noting a substantial influence of demographic covariates. However, an exception was observed for low HDL-C: the odds ratios increased after covariate adjustment, with the OR rising from 1.51 to 2.02 in the overweight group and from 2.36 to 3.35 in obesity group, suggesting possible negative confounding and a potentially attenuated association between BMI and low HDL-C in unadjusted models.

In the fully adjusted models (Model 3), obesity remained significantly associated with substantially higher odds of hypertension (OR = 7.08, 95% CI: 5.46–9.20), diabetes (OR = 3.25, 95% CI: 2.19–4.84), high TC (OR = 1.81, 95% CI: 1.38–2.35), high TG (OR = 4.80, 95% CI: 3.79–6.10), high LDL-C (OR = 3.07, 95% CI: 2.32–4.07), and low HDL-C (OR = 3.36, 95% CI: 2.67–4.25). Overweight was also significantly linked to increased odds of most CRFs, particularly hypertension, high TG, and low HDL-C; however, its association with diabetes was attenuated and became non-significant after full adjustment (OR = 1.26, 95% CI: 0.87–1.84). Association estimates remained stable across sequential models (Models 1 to 3), with robustness observed after controlling for sociodemographic, lifestyle, and occupational factors. Although the association estimates remained stable after adjusting for lifestyle and occupational factors in Model 3, these covariates were retained based on their potential roles as confounders or contextual modifiers in the BMI-CRF relationship. The minimal change in estimates further supports the robustness of our findings. Overall, obesity was associated with the greatest cardiometabolic burden, notably for hypertension and high TG.

### 3.3 RCS analysis

**Figure 2:**
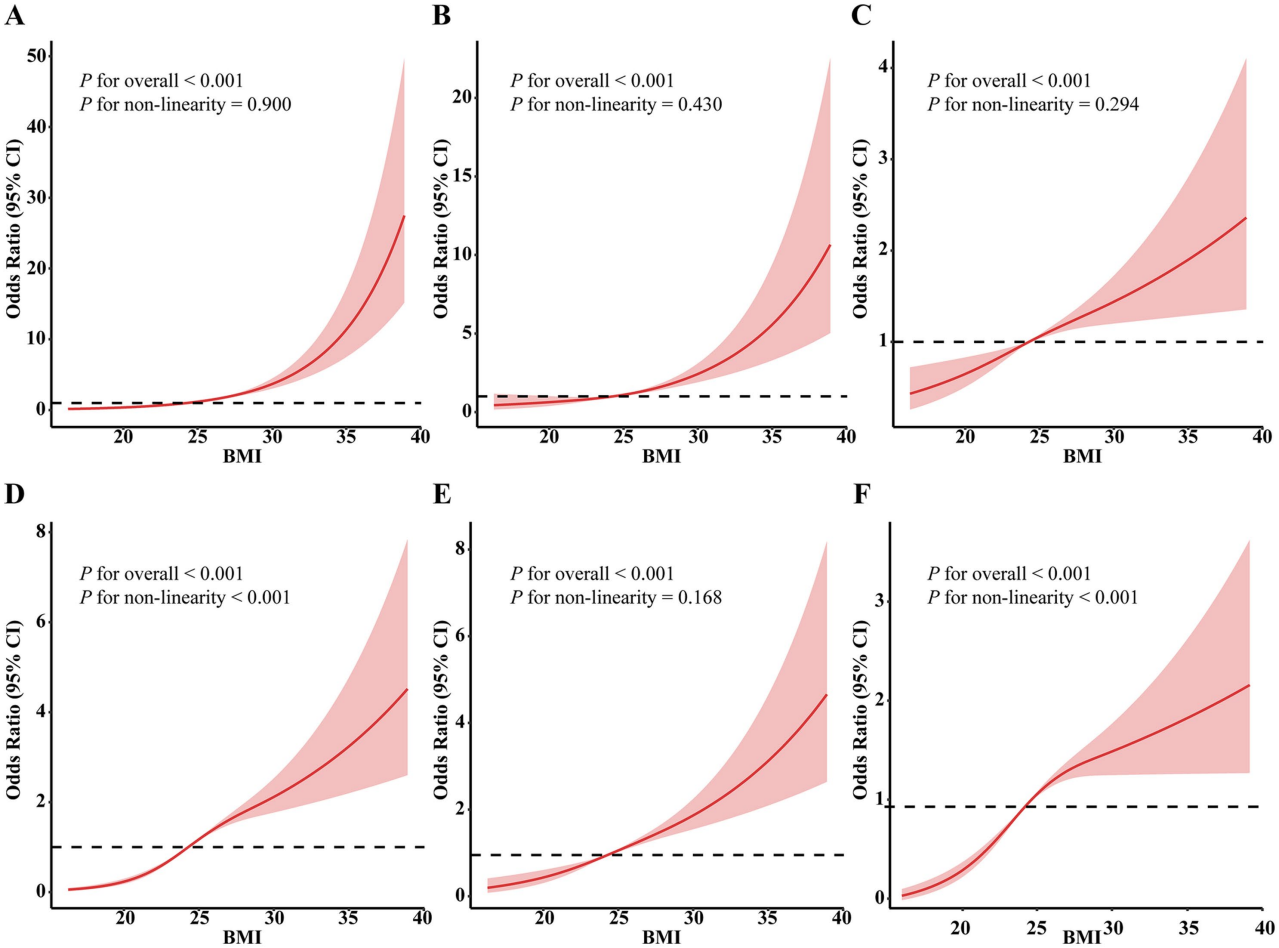
Dose–response pattern between body mass index and cardiometabolic risk factors using RCS models. **(A)** Hypertension, **(B)** diabetes, **(C)** high total cholesterol, **(D)** high triglycerides, **(E)** high low-density lipoprotein cholesterol, and **(F)** low high-density lipoprotein cholesterol. Restricted cubic spline models were constructed with the median BMI (24.28 kg/m^2^) as the reference value. BMI, body mass index; CI, confidence interval. Inflection points indicated in the curves are exploratory and derived from the fitted spline function; they do not represent predefined clinical thresholds.

According to the RCS analysis (Figure 2), significant overall associations were observed between BMI and the odds of hypertension, diabetes, high TC, high TG, high LDL-C, and low HDL-C (all *P* for overall < 0.001). Tests for nonlinearity showed no evidence of nonlinear associations for hypertension (*p* = 0.900), diabetes (*p* = 0.430), high TC (*p* = 0.294), and high LDL-C (*p* = 0.168). In contrast, high TG and low HDL-C exhibited significant nonlinear associations with BMI (both *p* < 0.001). A positive association was observed between BMI and the odds of these metabolic abnormalities across the full BMI range.

RCS analysis also revealed significant overall and non-linear associations between BMI and both high TG and low HDL-C. Exploratory inspection of the spline curves suggested local inflection points around BMI ≈ 26.84 kg/m^2^ for high TG and ≈29.25 kg/m^2^ for low HDL-C, corresponding to areas where the slope of the spline function increased more sharply. These values were not pre-specified cutoffs and should be interpreted solely as descriptive features of the modeled association. These results suggest that high TG levels begin to rise markedly around the threshold for overweight, while low HDL-C levels show a notable decrease at the threshold for obesity (Supplementary Tables S2, S3).

### 3.4 Subgroup and sensitivity analyses

**Figure 3:**
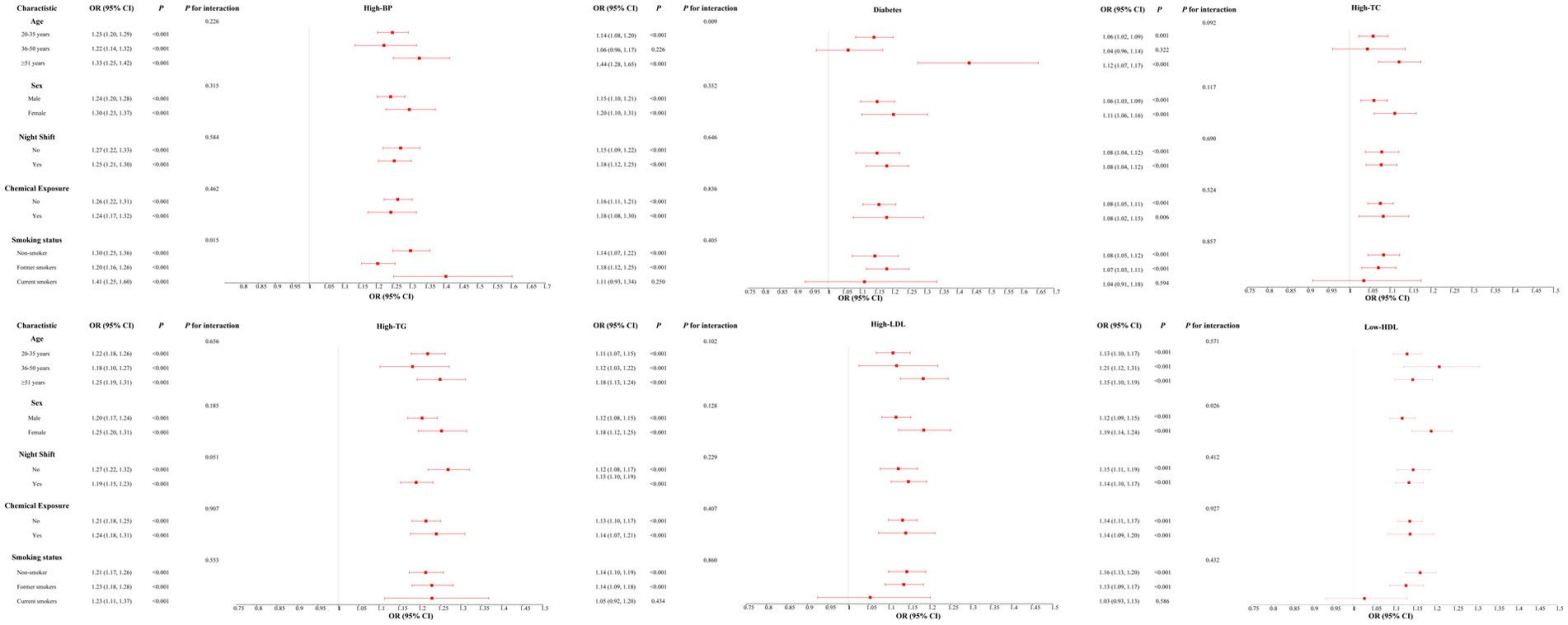
Stratified analyses of the associations between body mass index and six cardiometabolic risk factors by age, sex, chemical exposure, night-shift work, and smoking status. *p*-values for interaction were calculated from multiplicative interaction terms. ORs, odds ratios; CIs, confidence intervals.

As shown in Figure 3, stratified analyses indicated that the positive associations between BMI and each CRF were generally consistent across age, sex, night-shift work, smoking status, and chemical exposure. Significant interactions were observed for hypertension by smoking status (*P* for interaction = 0.015), diabetes by age group (*P* for interaction = 0.009), and low HDL-C by sex (*P* for interaction = 0.037). No significant interactions were detected for other stratification variables.

Sensitivity analyses supported the robustness of the observed associations: after multiple imputation for missing data (Supplementary Table S4) and when applying WHO BMI criteria (Supplementary Table S5), the direction and magnitude of estimates were broadly consistent with the main results.

## 4 Discussion

A key strength of our study lies in the robustness of its findings, which were confirmed through a sensitivity analysis using the WHO-recommended BMI cutoffs. This finding supports the appropriateness of our initial choice to use the Chinese-specific BMI standards, which are more tailored to the physiological characteristics of our study population. The consistent results across both classification systems highlight the relevance of screening for excess weight in this population, regardless of the classification criteria applied. Although occupational exposures were not the primary focus of this study, conducting the analysis within an oilfield workforce remains meaningful. The homogeneous work setting and shared lifestyle patterns of oilfield employees provide a stable context for examining BMI-CRF associations, reducing variability introduced by mixed occupational populations. Thus, the occupational background enhances—rather than weakens—the relevance of our findings to this specific workforce.

This study reaffirms BMI—as a practical, validated anthropometric measure of adiposity—as the primary exposure variable associated with multiple CRFs in an occupational population. The consistent and robust associations observed across overweight and obesity categories underscore BMI’s relevance for identifying individuals with metabolic abnormalities among Chinese oilfield workers.

Specifically, both overweight and obesity were significantly linked to higher odds of hypertension, diabetes, and dyslipidemia—including high TC, high TG, high LDL-C, and low HDL-C—with obesity being associated with the greatest overall cardiometabolic burden. Supporting these findings, RCS analyses revealed clear dose–response pattern between BMI and all CRFs, with evidence of potential nonlinear associations for high TG and low HDL-C. Collectively, these results highlight the relevance of considering excess adiposity in workplace health assessments to manage metabolic abnormalities in occupational populations.

Consistent with previous large-scale studies (21, 22), overweight and obesity were significantly associated with higher odds of most CRFs such as hypertension and dyslipidemia. However, the association between overweight and diabetes was attenuated and became non-significant after full adjustment, which contrasts with previous observational findings that reported positive associations between overweight and diabetes (23, 24). This discrepancy may be partly accounted for by and the fact that central adiposity—which is common in Asian populations—is more strongly associated with the odds of diabetes than BMI alone. A national cross-sectional study in Chinese adults demonstrated that abdominal obesity was independently linked to type 2 diabetes, regardless of BMI level (25), suggesting that central adiposity may capture additional metabolic burden beyond that reflected by BMI. While BMI remains a practical and widely used measure in population-based studies, incorporating waist circumference or other indicators of fat distribution may enhance association assessment in future research. Supporting this, sensitivity analysis using WHO BMI cutoffs recommended for Asian populations yielded slightly stronger association estimates for diabetes and high TG, underscoring concerns that universal BMI thresholds may inadequately capture ethnic differences in fat distribution and insulin sensitivity. Notably, the OR for low HDL-C increased after adjustment, suggesting negative confounding by lifestyle factors such as smoking and drinking, which may mask the underlying association in unadjusted models (26, 27). This emphasizes the importance of controlling for confounders when assessing dyslipidemia burden. However, these interpretations should be made cautiously, as the cross-sectional design precludes causal inference.

While prior research has predominantly focused on general or clinical populations, relatively few studies have examined the cardiometabolic implications of excess adiposity in occupational groups. This represents an important gap, as working populations—particularly those engaged in physically intensive, high-stress, or environmentally hazardous occupations—may carry distinct association profiles. For example, studies among coal miners, transportation workers, and industrial employees have reported variable associations between BMI and metabolic disorders, with some noting higher prevalence patterns, potentially related to factors such as shift work, chemical exposure, or chronic stress (28–30). However, findings remain inconsistent across occupational sectors and are often limited by small sample sizes or narrow exposure assessment. By analyzing a large, well-characterized sample of Chinese oilfield workers, our study provides context-specific confirmation of the well-documented associations between BMI and cardiometabolic risk factors. These findings underscore the importance of monitoring cardiometabolic profiles in industrial labor populations and offer occupation-specific descriptive data that may inform future surveillance research in similar high-risk workforces.

RCS demonstrated significant nonlinear associations between BMI and lipid abnormalities, particularly high TG and low HDL-C, with steeper increases in odds observed at higher BMI levels. These patterns are consistent with prior evidence showing that higher adiposity is associated with greater metabolic dysregulation, which are known to disproportionately impair lipid regulation in individuals with obesity (31). The BMI values at which the spline curves showed turning points were exploratory and data-driven, and therefore should not be interpreted as clinical thresholds. Confirmation in independent cohorts is needed.

In subgroup analyses, the associations between BMI and six CRFs were generally consistent across age, sex, night-shift work, smoking status, and chemical exposure, supporting the robustness of the main findings. Notably, stronger associations were observed for hypertension among current smokers, suggesting that smoking may be associated with obesity-related vascular odds through synergistic effects on systemic inflammation and endothelial injury (32). The BMI-diabetes association was more pronounced in older participants, which may reflect cumulative metabolic burden and which is consistent with age-related physiological changes reported in prior literature (33). In addition, sex significantly modified the association between BMI and low HDL-C, with male exhibiting a greater adverse association, consistent with evidence that sex hormones and fat distribution patterns influence HDL metabolism (34, 35). By contrast, no significant association modification was observed for night-shift work or chemical exposure. As multiple subgroup and interaction comparisons were performed, these results may be prone to chance findings and should therefore be interpreted cautiously as exploratory.

These findings should be interpreted with caution. The absence of statistically significant interactions does not necessarily imply that occupational exposures play a negligible role. Rather, the binary and self-reported nature of exposure variables likely introduced non-differential misclassification, which tends to bias interaction estimates toward the null. Therefore, the present study may not have had sufficient resolution to detect the potential contribution of occupational hazards. More refined exposure assessment—incorporating quantitative intensity, duration, and cumulative dose—is needed to clarify how occupational factors may jointly influence metabolic health alongside adiposity. Nevertheless, given prior evidence linking chemical exposures to metabolic disturbances, further longitudinal research is warranted to clarify potential synergistic effects between adiposity and workplace hazards (36). Although ethnic differences in adiposity and fat distribution may influence metabolic risk in China, our subgroup analyses were limited to age, sex, chemical exposure, night-shift work, and smoking status. Future studies with larger and more diverse samples are needed to examine potential ethnic heterogeneity and to develop culturally tailored workplace health strategies.

This study has several limitations that should be acknowledged. First, the cross-sectional design precludes any inference about causal relationships between BMI and CRFs. Additionally, because several CRFs in this population are relatively common (>30%), the ORs estimated from logistic regression may overestimate the true prevalence ratios and should be interpreted as measures of association rather than direct risk ratios. Future studies using log-binomial or modified Poisson regression models could provide more accurate estimates of prevalence ratios. Longitudinal studies are also needed to confirm temporal associations and clarify potential causal pathways. Second, a total of 23.7% of participants were excluded from the primary analysis due to missing covariate information, although missingness for the primary exposure (BMI, 0.05%) and outcomes (CRFs, 2.3%) was minimal. Complete-case analysis was considered appropriate as the main analytic approach, and sensitivity analyses using multiple imputation for missing covariates yielded broadly consistent results. Third, BMI cannot differentiate fat from lean mass or fully capture visceral fat—which may be more relevant to metabolic risk, especially in Asian populations. However, it remains a practical and standardized measure of adiposity in large occupational cohorts. Future studies could integrate additional indicators to better assess fat distribution. Despite its limitations, our findings support the relevance of BMI as an indicator for identifying individuals with cardiometabolic abnormalities. Fourth, although we adjusted for a comprehensive set of covariates, residual confounding due to unmeasured or imprecisely measured variables cannot be ruled out. In particular, key factors such as detailed dietary intake, psychosocial stress, and genetic susceptibility were not captured by the health examination system. Moreover, several occupational exposures—including chemical exposure, night-shift work, noise, and dust—were obtained through self-reported binary classifications without quantitative information on duration, frequency, or intensity. This may have reduced the precision of subgroup analyses and limited our ability to evaluate dose–response effects. Finally, the study population was drawn from a single oilfield-affiliated hospital in northwest China, limiting the generalizability of our findings to other occupational sectors or the general Chinese population. The use of Chinese-specific BMI criteria may further constrain comparability with studies based on WHO standards. Therefore, caution is warranted when extrapolating our results to non-Asian populations or settings with different demographic and occupational characteristics.

The findings of this study should be interpreted with caution, particularly given the limitations of its cross-sectional design. First, although the results underscore the importance of monitoring excess weight in occupational populations—where work routines and environmental conditions may influence metabolic health—future longitudinal studies are required to clarify temporal relationships. Second, the stronger associations observed when applying WHO BMI thresholds suggest that current national criteria may underestimate cardiometabolic susceptibility in certain subgroups, but confirmation in prospective and nationally representative cohorts is necessary. Third, although an interaction between BMI and chemical exposure was detected, the absence of consistent associations across occupational exposures indicates that further mechanistic and longitudinal evidence is needed before drawing firm conclusions. Overall, rather than supporting immediate intervention strategies, these findings primarily highlight the need for continued surveillance and future cohort studies to better understand how adiposity and workplace factors jointly relate to cardiometabolic health in industrial workforces.

## 5 Conclusion

In this occupational population, BMI was independently associated with increased odds of hypertension, diabetes, and dyslipidemia, with obesity exhibiting the strongest associations across CRFs. The observed dose–response pattern and subgroup differences highlight the importance of excess adiposity as a modifiable factor, underscoring the need for targeted screening and prevention strategies in industrial workforces.

## Data Availability

The original contributions presented in the study are included in the article/Supplementary material, further inquiries can be directed to the corresponding author/s.

## References

[1] Sørensen TIA. Forecasting the global obesity epidemic through 2050. Lancet. (2025) 405:756–7. doi: 10.1016/S0140-6736(25)00260-0, 40049184

[2] GBD 2021 Adult BMI Collaborators. Global, regional, and national prevalence of adult overweight and obesity, 1990-2021, with forecasts to 2050: a forecasting study for the global burden of disease study 2021. Lancet. (2025) 405:813–38. doi: 10.1016/S0140-6736(25)00355-1, 40049186 PMC11920007

[3] NCD Risk Factor Collaboration (NCD-RisC). Worldwide trends in underweight and obesity from 1990 to 2022: a pooled analysis of 3663 population-representative studies with 222 million children, adolescents, and adults. Lancet. (2024) 403:1027–50. doi: 10.1016/S0140-6736(23)02750-2, 38432237 PMC7615769

[4] Center For Cardiovascular Diseases The Writing Committee Of The Report On Cardiovascular Health And Diseases In China N. Report on cardiovascular health and diseases in China 2023: an updated summary. Biomed Environ Sci. (2024) 37:949–92. doi: 10.3967/bes2024.162, 39401992

[5] Sun X Yan AF Shi Z Zhao B Yan N Li K . Health consequences of obesity and projected future obesity health burden in China. Obesity. (2022) 30:1724–51. doi: 10.1002/oby.23472, 36000246

[6] Powell-Wiley TM Poirier P Burke LE Després J-P Gordon-Larsen P Lavie CJ . Obesity and cardiovascular disease: a scientific statement from the American Heart Association. Circulation. (2021) 143:e984–e1010. doi: 10.1161/CIR.0000000000000973, 33882682 PMC8493650

[7] Pan X-F Wang L Pan A. Epidemiology and determinants of obesity in China. Lancet Diabetes Endocrinol. (2021) 9:373–92. doi: 10.1016/S2213-8587(21)00045-0, 34022156

[8] GBD 2019 Risk Factors Collaborators. Global burden of 87 risk factors in 204 countries and territories, 1990-2019: a systematic analysis for the global burden of disease study 2019. Lancet. (2020) 396:1223–49. doi: 10.1016/S0140-6736(20)30752-2, 33069327 PMC7566194

[9] Poirier P Giles TD Bray GA Hong Y Stern JS Pi-Sunyer FX . Obesity and cardiovascular disease: pathophysiology, evaluation, and effect of weight loss: an update of the 1997 American Heart Association scientific statement on obesity and heart disease from the obesity Committee of the Council on nutrition, physical activity, and metabolism. Circulation. (2006) 113:898–918. doi: 10.1161/CIRCULATIONAHA.106.171016, 16380542

[10] Yusuf S Hawken S Ounpuu S Dans T Avezum A Lanas F . Effect of potentially modifiable risk factors associated with myocardial infarction in 52 countries (the INTERHEART study): case-control study. Lancet. (2004) 364:937–52. doi: 10.1016/S0140-6736(04)17018-9, 15364185

[11] Meng M Guo Y Kuang Z Liu L Cai Y Ni X. Risk of stroke among different metabolic obesity phenotypes: a systematic review and Meta-analysis. Front Cardiovasc Med. (2022) 9:844550. doi: 10.3389/fcvm.2022.844550, 35548434 PMC9081493

[12] Wang X Dong J Du Z Jiang J Hu Y Qin L . Risk of heart failure between different metabolic states of health and weight: a Meta-analysis of cohort studies. Nutrients. (2022) 14:5223. doi: 10.3390/nu14245223, 36558382 PMC9785251

[13] Wei D González-Marrachelli V Melgarejo JD Liao C-T Hu A Janssens S . Cardiovascular risk of metabolically healthy obesity in two european populations: prevention potential from a metabolomic study. Cardiovasc Diabetol. (2023) 22:82. doi: 10.1186/s12933-023-01815-6, 37029406 PMC10082537

[14] Nazare JA Smith JD Borel AL Haffner SM Balkau B Ross R . Ethnic influences on the relations between abdominal subcutaneous and visceral adiposity, liver fat, and cardiometabolic risk profile: the international study of prediction of intra-abdominal adiposity and its relationship with Cardiometabolic risk/intra-abdominal adiposity. Am J Clin Nutr. (2012) 96:714–26. doi: 10.3945/ajcn.112.035758 22932278

[15] Zhou BF. Cooperative Meta-Analysis Group of the Working Group on Obesity in China. Predictive values of body mass index and waist circumference for risk factors of certain related diseases in Chinese adults--study on optimal cut-off points of body mass index and waist circumference in Chinese adults. Biomed Environ Sci. (2002) 15:83–96. 12046553

[16] Li J-J Liu H-H Li S. Landscape of cardiometabolic risk factors in Chinese population: a narrative review. Cardiovasc Diabetol. (2022) 21:113. doi: 10.1186/s12933-022-01551-3, 35729555 PMC9215083

[17] Regression modeling strategies: With applications to linear models, logistic and ordinal regression, and survival analysis. ResearchGate. Cham, Switzerland: Springer International Publishing. (2015), 211–258. Available online at: https://www.researchgate.net/publication/303105948_Regression_Modeling_Strategies_With_Applications_to_Linear_Models_Logistic_and_Ordinal_Regression_and_Survival_Analysis (Accessed August 1, 2025)

[18] Heindel JJ Howard S Agay-Shay K Arrebola JP Audouze K Babin PJ . Obesity II: establishing causal links between chemical exposures and obesity. Biochem Pharmacol. (2022) 199:115015. doi: 10.1016/j.bcp.2022.115015, 35395240 PMC9124454

[19] Stratakis N Rock S La Merrill MA Saez M Robinson O Fecht D . Prenatal exposure to persistent organic pollutants and childhood obesity: a systematic review and meta-analysis of human studies. Obes Rev. (2022) 23:e13383. doi: 10.1111/obr.13383, 34766696 PMC9512275

[20] El-Metwally A Fatani F Binhowaimel N Al Khateeb BF Al Kadri HM Alshahrani A . Effect modification by age and gender in the correlation between diabetes mellitus, hypertension, and obesity. J Prim Care Community Health. (2023) 14:21501319231220234. doi: 10.1177/21501319231220234, 38140745 PMC10748554

[21] Zhu J Zhang Y Wu Y Xiang Y Tong X Yu Y . Obesity and dyslipidemia in Chinese adults: a cross-sectional study in Shanghai, China. Nutrients. (2022) 14:2321. doi: 10.3390/nu14112321, 35684121 PMC9183153

[22] Jayedi A Rashidy-Pour A Khorshidi M Shab-Bidar S. Body mass index, abdominal adiposity, weight gain and risk of developing hypertension: a systematic review and dose-response meta-analysis of more than 2.3 million participants. Obes Rev. (2018) 19:654–67. doi: 10.1111/obr.12656, 29334692

[23] Yu H-J Ho M Liu X Yang J Chau PH Fong DYT. Incidence and temporal trends in type 2 diabetes by weight status: a systematic review and meta-analysis of prospective cohort studies. J Glob Health. (2023) 13:04088. doi: 10.7189/jogh.13.04088, 37651631 PMC10471153

[24] Wang X Wu Y Wang Y Zhou J Liu T. Relationship between metabolically healthy overweight/obesity and risk of type 2 diabetes in different ethnicity: a prospective cohort study in Southwest China. BMC Public Health. (2024) 24:2798. doi: 10.1186/s12889-024-20254-w, 39396945 PMC11472488

[25] Zhang S Li W Jia X Zhang J Jiang H Wang L . Association of obesity profiles with type 2 diabetes in Chinese adults: findings from the China health and nutrition survey. Front Nutr. (2022) 9:922824. doi: 10.3389/fnut.2022.922824, 36176634 PMC9513418

[26] Ghodeshwar GK Dube A Khobragade D. Impact of lifestyle modifications on cardiovascular health: a narrative review. Cureus. 15:e42616. doi: 10.7759/cureus.42616 PMC10460604 37641769

[27] Liu C Dhindsa D Almuwaqqat Z Ko Y-A Mehta A Alkhoder AA . Association between high-density lipoprotein cholesterol levels and adverse cardiovascular outcomes in high-risk populations. JAMA Cardiol. (2022) 7:672–80. doi: 10.1001/jamacardio.2022.0912, 35583863 PMC9118072

[28] Fassio F Bussa M Oddone E Ferraro OE Puci MV Morandi A . Health status of petrochemical workers: a narrative review. G Ital Med Lav Ergon. (2022) 44:51–8. doi: 10.4081/gimle.581, 36346299

[29] Yang X Di W Zeng Y Liu D Han M Qie R . Association between shift work and risk of metabolic syndrome: a systematic review and meta-analysis. Nutr Metab Cardiovasc Dis. (2021) 31:2792–9. doi: 10.1016/j.numecd.2021.06.007, 34332862

[30] Sit G Letellier N Iwatsubo Y Goldberg M Leynaert B Nadif R . Occupational exposures to organic solvents and asthma symptoms in the CONSTANCES cohort. Int J Environ Res Public Health. (2021) 18:9258. doi: 10.3390/ijerph18179258, 34501848 PMC8431091

[31] Li M Zhang W Zhang M Li L Wang D Yan G . Nonlinear relationship between untraditional lipid parameters and the risk of prediabetes: a large retrospective study based on Chinese adults. Cardiovasc Diabetol. (2024) 23:12. doi: 10.1186/s12933-023-02103-z, 38184606 PMC10771669

[32] Aragiannis D Kasiakogias A Iliakis P Sagris M Tatakis FP Manta E . The combined effect of smoking and obesity on hypertension: implications for clinical management. Curr Hypertens Rev. (2025) 21:2–14. doi: 10.2174/0115734021351026250126165154, 39950475

[33] Kolb H Kempf K Martin S. Insulin and aging - a disappointing relationship. Front Endocrinol. (2023) 14:1261298. doi: 10.3389/fendo.2023.1261298, 37854186 PMC10579801

[34] van Oortmerssen JAE Mulder JWCM Kavousi M Roeters van Lennep JE. Lipid metabolism in women: A review. Atherosclerosis. (2025) 405:119213. doi: 10.1016/j.atherosclerosis.2025.119213 40300433

[35] Holven KB van Roeters Lennep J. Sex differences in lipids: a life course approach. Atherosclerosis. (2023) 384:117270. doi: 10.1016/j.atherosclerosis.2023.117270, 37730457

[36] Denic-Roberts H Engel LS Buchanich JM Miller RG Talbott EO Thomas DL . Risk of longer-term endocrine and metabolic conditions in the Deepwater horizon oil spill coast guard cohort study - five years of follow-up. Environ Health. (2025) 24:12. doi: 10.1186/s12940-025-01164-9, 40121483 PMC11929317

