# Supplementary material for: Overweight or obesity and their association with cardiometabolic risk factors among oilfield workers in Chinese population: a cross-sectional study
Source: Front Public Health. 2026 Feb 16;14:1658235. doi: 10.3389/fpubh.2026.1658235 (PMC12950669; doi:10.3389/fpubh.2026.1658235)
Supplement: Supplementary file 1 [file Data_Sheet_1.pdf]

Supplementary Material

Table S1. Category and definition of covariates.

Table S2. Analysis of the threshold effect of BMI on high triglycerides (high TG)

Table S3. Analysis of the threshold effect of BMI on low high-density lipoprotein (low HDL)

Table S4. Associations between body mass index and cardiometabolic risk factors, employing multiple imputations for covariates.

Table S5. Associations between body mass index and cardiometabolic risk factors, performing analyses based on WHO criteria for body mass index.

Table S1. Category and definition of covariates.

Variable	Category and definition
Smoking status	Smoking status is categorized into three groups: current smokers, former smokers, and non-smoker. Current smokers refer to smoking at least one cigarette daily for over six months, while former smokers indicate having quit for more than half a years.
Drinking status	Drinking status is divided into three categories: current drinkers, former drinkers, and non-drinker. Current drinkers are defined as consuming alcohol at least twice weekly, with a minimum intake of 50 ml, maintained for over six months. Former drinkers refer to abstaining from alcohol for more than half a year.
Exercise	Exercise is evaluated in terms of the metabolic equivalent of task (MET), taking into account the duration, frequency, and intensity of vigorous and moderate exercise during leisure time each week.<br>Inactive: <600 MET-min/week.<br>Active: ≥600 MET-min/week.
Chemical Exposure	Chemical Exposure refers to operations where workers may be exposed to harmful chemical substances, such as benzene, toluene, xylene, and hydrogen sulfide.
Noise exposure	Noise exposure refers to sounds in the workplace that can damage hearing and health, specifically when the noise level reaches 80 dB(A) or higher for 8 hours per day or 40 hours per week.
Dust exposure	Dust exposure occurs when workers come into contact with occupational dust during work.
Shift work	Shift work is defined as the regular rotation of individuals to work outside the hours of 8:00 AM to 5:00 PM for a minimum duration of one year.

Table S2. Analysis of the threshold effect of BMI on high triglycerides (high TG)

Outcome		OR (95% CI)	P-value
**Model 1**		1.22 (1.19, 1.25)	<0.001
**Model 2**	Inflection Point	26.84	
	< 26.84	1.34 (1.28, 1.41)	<0.001
	≥ 26.84	1.08 (1.01,1.15)	0.022
**P-value for Likelihood**			<0.001

Model 1: Fitting model by standard linear regression; Model 2: Fitting model by two-piecewise linear regression.
BMI, Body Mass Index; OR, odds ratio; CI, confidence interval; TG, Triglyceride.

Table S3. Analysis of the threshold effect of BMI on low high-density lipoprotein (low HDL)

Outcome		OR (95% CI)	P-value
**Model 1**		1.14 (1.11,1.17)	<0.001
**Model 2**	Inflection Point	29.25	
	< 29.25	1.21 (1.17.1.25)	<0.001
	≥ 29.25	0.93 (0.85,1.02)	0.131
**P-value for Likelihood**			<0.001

Model 1: Fitting model by standard linear regression; Model 2: Fitting model by two-piecewise linear regression.
BMI, Body Mass Index; OR, odds ratio; CI, confidence interval; HDL-C, High-Density Lipoprotein.

Table S4. Associations between body mass index and cardiometabolic risk factors, employing multiple imputations for covariates.

Variable	Model 1		Model 2		Model 3	
	OR (95% CI)	*P* value	OR (95% CI)	*P* value	OR (95% CI)	*P* value
**Hypertension**						
Normal	1.00 (Reference)		1.00 (Reference)		1.00 (Reference)	
Overweight	2.70 (2.25, 3.23)	<0.001	2.20 (1.82, 2.65)	<0.001	2.22 (1.82, 2.71)	<0.001
Obesity	6.96 (5.65, 8.59)	<0.001	6.55 (5.25, 8.19)	<0.001	6.82 (5.41, 8.63)	<0.001
P for trend		<0.001		<0.001		<0.001
**Diabetes**						
Normal	1.00 (Reference)		1.00 (Reference)		1.00 (Reference)	
Overweight	1.58 (1.18, 2.12)	0.002	1.19 (0.88, 1.62)	0.258	1.22 (0.89, 1.69)	0.221
Obesity	2.98 (2.16, 4.11)	<0.001	2.53 (1.80, 3.53)	<0.001	2.65 (1.86, 3.78)	<0.001
P for trend		<0.001		<0.001		<0.001
**High TC**						
Normal	1.00 (Reference)		1.00 (Reference)		1.00 (Reference)	
Overweight	1.43 (1.20, 1.71)	<0.001	1.31 (1.09, 1.58)	0.004	1.28 (1.06, 1.56)	0.012
Obesity	1.87 (1.50, 2.33)	<0.001	1.74 (1.38, 2.19)	<0.001	1.68 (1.32, 2.13)	<0.001
P for trend		<0.001		<0.001		<0.001
**High TG**						
Normal	1.00 (Reference)		1.00 (Reference)		1.00 (Reference)	
Overweight	3.66 (3.17, 4.24)	<0.001	2.82 (2.42, 3.28)	<0.001	2.78 (2.38, 3.27)	<0.001
Obesity	5.90 (4.86, 7.19)	<0.001	4.55 (3.71, 5.58)	<0.001	4.52 (3.66, 5.60)	<0.001
P for trend		<0.001		<0.001		<0.001
**High LDL**-C						
Normal	1.00 (Reference)		1.00 (Reference)		1.00 (Reference)	
Overweight	2.04 (1.67, 2.49)	<0.001	1.80 (1.46, 2.22)	<0.001	1.71 (1.38, 2.13)	<0.001
Obesity	3.50 (2.77, 4.41)	<0.001	3.07 (2.41, 3.91)	<0.001	3.02 (2.35, 3.88)	<0.001
P for trend		<0.001		<0.001		<0.001
**Low HDL**-C						
Normal	1.00 (Reference)		1.00 (Reference)		1.00 (Reference)	
Overweight	1.65 (1.43, 1.91)	<0.001	2.21 (1.89, 2.59)	<0.001	2.15 (1.83, 2.53)	<0.001
Obesity	2.38 (1.98, 2.87)	<0.001	3.37 (2.75, 4.12)	<0.001	3.44 (2.79, 4.24)	<0.001
P for trend		<0.001		<0.001		<0.001

Model 1, no covariate was adjusted. Model 2, adjusted for age, sex, education, marital status, income, ethnicity. Model 3, adjusted for age, sex, education, marital status, income, ethnicity, exercise,smoking status, drinking status, night shift, chemical exposure, noise exposure and dust exposure. BMI, Body Mass Index; CRFs, Cardiometabolic Risk Factors; OR, odds ratio; CI, confidence interval; TC, total cholesterol; TG, Triglyceride; LDL-C, Low-Density Lipoprotein; HDL-C, High-Density Lipoprotein.

Table S5. Associations between body mass index and cardiometabolic risk factors, performing analyses based on WHO-recommended BMI cut-off points for Asian populations.

Variable	Model 1		Model 2		Model 3	
	OR (95% CI)	*P* value	OR (95% CI)	*P* value	OR (95% CI)	*P* value
**Hypertension**						
Normal	1.00 (Reference)		1.00 (Reference)		1.00 (Reference)	
Overweight	2.51 (2.06, 3.07)	<0.001	2.03 (1.65, 2.50)	<0.001	1.99 (1.60, 2.49)	<0.001
Obesity	7.33 (5.90, 9.16)	<0.001	6.44 (5.11, 8.14)	<0.001	6.54 (5.13, 8.37)	<0.001
P for trend		<0.001		<0.001		<0.001
**Diabetes**						
Normal	1.00 (Reference)		1.00 (Reference)		1.00 (Reference)	
Overweight	1.76 (1.27, 2.45)	<0.001	1.35 (0.97, 1.91)	0.081	1.41 (0.99, 2.03)	0.061
Obesity	3.47 (2.47, 4.93)	<0.001	2.78 (1.95, 4.02)	<0.001	2.95 (2.02, 4.34)	<0.001
P for trend		<0.001		<0.001		<0.001
**High TC**						
Normal	1.00 (Reference)		1.00 (Reference)		1.00 (Reference)	
Overweight	1.66 (1.38, 2.02)	<0.001	1.54 (1.26, 1.88)	<0.001	1.57 (1.28, 1.93)	<0.001
Obesity	2.13 (1.70, 2.67)	<0.001	1.98 (1.56, 2.50)	<0.001	1.88 (1.47, 2.41)	<0.001
P for trend		<0.001		<0.001		<0.001
**High TG**						
Normal	1.00 (Reference)		1.00 (Reference)		1.00 (Reference)	
Overweight	3.86 (3.31, 4.52)	<0.001	3.03 (2.58, 3.57)	<0.001	3.06 (2.58, 3.63)	<0.001
Obesity	7.13 (5.87, 8.69)	<0.001	5.30 (4.33, 6.51)	<0.001	5.28 (4.27, 6.55)	<0.001
P for trend		<0.001		<0.001		<0.001
**High LDL-C**						
Normal	1.00 (Reference)		1.00 (Reference)		1.00 (Reference)	
Overweight	2.26 (1.81, 2.84)	<0.001	2.01 (1.59, 2.54)	<0.001	1.99 (1.56, 2.55)	<0.001
Obesity	4.07 (3.18, 5.23)	<0.001	3.51 (2.71, 4.56)	<0.001	3.45 (2.64, 4.53)	<0.001
P for trend		<0.001		<0.001		<0.001
**Low HDL-C**						
Normal	1.00 (Reference)		1.00 (Reference)		1.00 (Reference)	
Overweight	1.72 (1.48, 2.00)	<0.001	2.28 (1.94, 2.69)	<0.001	2.23 (1.88, 2.65)	<0.001
Obesity	2.52 (2.10, 3.03)	<0.001	3.76 (3.08, 4.61)	<0.001	3.81 (3.09, 4.71)	<0.001
P for trend		<0.001		<0.001		<0.001

Model 1, no covariate was adjusted. Model 2, adjusted for age, sex, education, marital status, income, ethnicity. Model 3, adjusted for age, sex, education, marital status, income, ethnicity, exercise,smoking status, drinking status, night shift, chemical exposure, noise exposure and dust exposure. BMI, Body Mass Index; CRFs, Cardiometabolic Risk Factors; OR, odds ratio; CI, confidence interval; TC, total cholesterol; TG, Triglyceride; LDL-C, Low-Density Lipoprotein; HDL-C, High-Density Lipoprotein.

According to WHO recommendations for Asian populations, BMI values of ≥23.0 kg/m² and ≥27.5 kg/m² are suggested as alternative cut-off points for identifying overweight and obesity, respectively, due to higher cardiometabolic risks at lower BMI levels compared to Western populations.
